# Superconducting UCN Polarizer for a New EDM Spectrometer

**DOI:** 10.6028/jres.110.022

**Published:** 2005-06-01

**Authors:** A. Serebrov, M. Lasakov, A. Fomin, P. Geltenbort, A. Murashkin, I. Krasnoshekova, Yu. Rudnev, A. Vasiliev

**Affiliations:** St. Petersburg Nuclear Physics Institute, Gatchina, Russia; Institut Max von Laue–Paul Langevin, Grenoble, France; St. Petersburg Nuclear Physics Institute, Gatchina, Russia

**Keywords:** polarization, ultracold neutrons

## Abstract

A test experiment has shown that the number of ultracold neutrons (UCN) of one polarization state, transmitted through a 100 μm Al foil when placed in a 5 T magnetic field, is greater by 3.8 times. The increased transmission is due to the higher velocity of the UCN passing through the foil.

## 1. Introduction

There are two possible ways to obtain polarized UCN for the electric dipole moment (EDM) experiment: either one uses magnetized ferromagnetic layers on thin foils [1] or one uses strong magnetic fields of the order of 5 T.

We propose to use a superconducting (SC) solenoid polarizer on the fill lines of the EDM spectrometer. The advantages of such a choice are: 1) the possibility to obtain fully polarized UCN as compared to about 85 % polarization using magnetized foils; 2) the possibility to place the vacuum separation foil in the high magnetic field region.

Separation foils are needed between the UCN source volume containing the solid deuterium and the EDM volume with the high voltage. A test experiment has recently shown that the number of UCN of one polarization state transmitted through a 100 μm Al foil when placed in a 5 T magnetic field is greater by 3.8 times. The increased transmission is due to the higher velocity of the UCN passing through the foil.

The superconducting solenoids will be equipped with ARMCO return yokes in order to suppress the stray fields that might influence the EDM measurements. Due to the fact that the magnetic field of the SC magnets, once switched on, will be very stable over typical measurement times, its influence on the EDM experiment is only static and can be compensated for. In our test experiment on foil transmission at the Institut Max von Laue–Paul Langevin (ILL), the possibility to carry out the present RAL-Sussex-ILL EDM experiment together with an even unshielded SC solenoid nearby (about 4 m distance) has been demonstrated.

As it is shown in the calculation, a cylindrical magnetic shield made of ARMCO with a diameter of 700 mm and a thickness of 100 mm can suppress the magnetic field in the EDM spectrometer down to 50 μT.

## 2. The Scheme of the Experiment

**Fig. 1:**
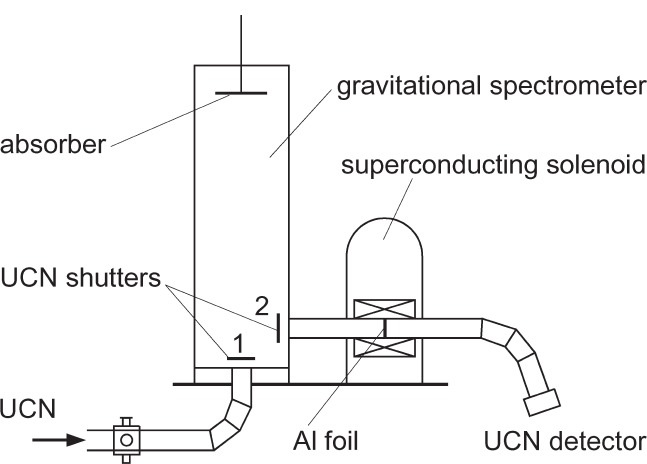
The scheme of the experimental setup.

The scheme of the experiment, which was recently performed at ILL, Grenoble, is shown in Fig. 1. UCN from the ILL turbine filled a Be coated gravitational spectrometer volume. After closing the shutter 1, a well defined UCN spectrum was formed in the spectrometer over 100 s by means of a moveable absorber. The spectrum was very similar to the one that will be obtained in the UCN storage vessel of the PSI UCN source, which is presently under construction.

When the shutter 2 was opened, UCN were counted in the UCN detector. Various cases were studied: with and without an Al foil, with and without a magnetic field. All four possible cases were studied with the UCN absorber at various positions in order to obtain the energy dependence of the transmission.

## 3. Experimental Results

**Fig. 2:**
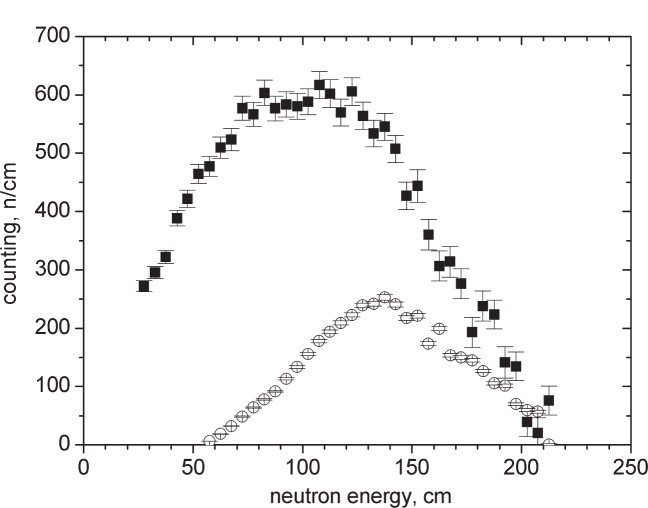
Differential UCN spectrum for Al foil (100 μm). -■- is the case when the magnetic field is switched on to 5 T. -○- is the case when the magnetic field is switched off (the counting rate is scaled by a factor of two in order to account for the fact that without the field, the two polarization components are present while for the solenoid switched on, only one polarization component is transmitted).

**Fig. 3:**
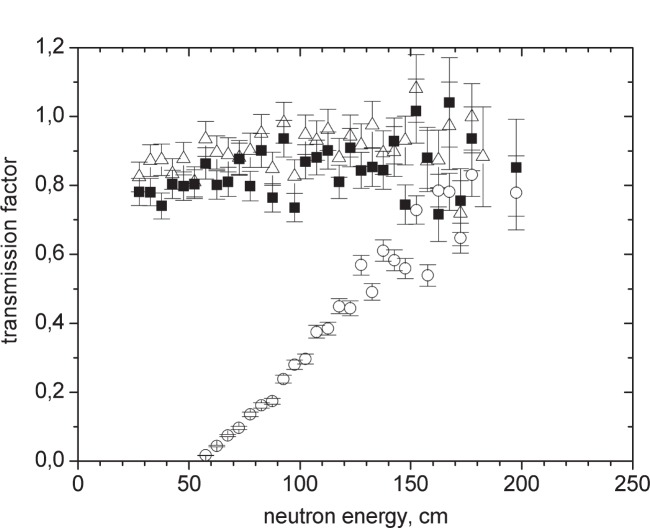
Absolute transmission of foils with and without the magnetic field as a function of UCN energy (height). -■- is the case with an Al foil (100 μm) and with the magnetic field switched on. -○- is the case with an Al foil (100 μm) and with the magnetic field switched off. -Δ- is the case with a Zr foil (50 μm) and with the magnetic field switched on.

Figure 2 shows the differential UCN spectrum, as seen by the UCN detector, with the magnetic field switched on to 5 T and the differential UCN spectrum with the magnetic field switched off divided by factor of two because it is an unpolarized beam with two spin components. For a 100 μm thick Al foil the integral number of polarized UCN with the SC magnet switched on is 3.8 times greater than the integral number of UCN with one spin component with the magnet switched off. UCN of one polarization component get accelerated in the magnetic field gradient, and have a longitudinal velocity of more than 7.6 m/s for the case where the 5 T field is at the foil position. As a result, these neutrons more easily penetrate the Al potential barrier and pass through the foil with considerably smaller losses. Figure 3 shows the transmission probability as a function of UCN energy outside the solenoid. This probability is determined by the ratio of count rates with and without the foil. In the case where the magnet is switched on, the transmission is only weakly dependent on the spectrum. For the 100 μm Al foil, the transmission is larger than 80 %. For a 50 μm Zr foil (which can be used for separating the vacuum in the same way as 100 μm Al), the transmission is about 90 %.

Thus the usage of a SC solenoid allows one to obtain polarized UCN and to increase the density of polarized UCN by factor of 3.8. In the cases when polarization of UCN is not needed in the experiment, the factor of the UCN density increase is about two times. It is important also for the experiments with unpolarized UCN.
